# Health education in the prevention and management of infectious diarrhea in childhood: A systematic review of randomized controlled trials

**DOI:** 10.1371/journal.pntd.0014442

**Published:** 2026-07-23

**Authors:** Arthur Santos Lima, Pedro Silva Dantas, Ana Beatriz Cazé-Cerón, Leonardo Paiva Farias, Natalia Machado Tavares, Viviane Sampaio Boaventura

**Affiliations:** 1 Fundação Oswaldo Cruz (Fiocruz), Instituto Gonçalo Moniz, Salvador, Bahia, Brazil; 2 Instituto Nacional de Ciência e Tecnologia em Saúde Digital (DigiSaúde-INCT), Salvador, Bahia, Brazil; 3 Instituto Nacional de Ciência e Tecnologia, Instituto de Investigação em Imunologia (INCT-iii), São Paulo, Brazil; 4 Instituto Nacional de Ciência e Tecnologia em Mucosa e Pele, Belo Horizonte, Brazil; 5 Laboratório de Medicina e Saúde Pública de Precisão, Fundação Oswaldo Cruz (Fiocruz), Salvador, Bahia, Brazil; 6 Faculdade de Medicina da Bahia, Universidade Federal da Bahia, Salvador, Bahia, Brazil; UFSJ: Universidade Federal de Sao Joao del-Rei, BRAZIL

## Abstract

**Background:**

Childhood diarrhea remains a leading cause of morbidity and mortality in low- and middle-income countries. Educational interventions targeting caregivers may promote preventive behaviors, but the overall evidence base has not yet been comprehensively synthesized. This systematic review evaluates the effectiveness and types of community-based educational interventions in caregivers of children under five years of age to prevent diarrhea.

**Methods and findings:**

We systematically searched MEDLINE/PubMed, EMBASE, Cochrane Library and LILACS with relevant keywords and MeSH terms from inception to December 2025. Of 1,917 records screened, 15 controlled trials (1985–2018) from Asia, Africa, and Latin America met inclusion criteria. Interventions included home visits, audiovisual materials, group education sessions, and behavior change communication (BCC) strategies. The primary outcomes were diarrhea incidence and prevention and hygiene-related behavioral changes. Risk of bias was assessed using RoB-2 for all studies. Most interventions reported positive outcomes, such as reduced diarrhea incidence and improved caregiver knowledge and enhanced hygiene behaviors. Multicomponent approaches, especially those combining home visits with audiovisual tools and BCC, were particularly effective. However, heterogeneity in follow-up periods, population characteristics, and settings limited comparability. Several studies had high or unclear risk of bias, often due to inadequate reporting of randomization or lack of blinding.

**Conclusion:**

Community-based educational strategies show potential for reducing childhood diarrhea, particularly when tailored to local contexts and combined multicomponent approaches. Future high-quality, standardized RCTs are needed to build a more robust evidence base and guide policy and program development.

## Author summary

Diarrhea remains a leading cause of death among children under five worldwide, primarily driven by inadequate sanitation and poor hygiene. While educating caregivers on hygiene and prevention is a common strategy, its effectiveness can vary widely depending on how it is delivered and the local context. In this systematic review, we analyzed 15 clinical trials to evaluate the impact of community-based health education programs on childhood diarrhea. We found that educational interventions—such as home visits, instructional videos, and community group sessions—can significantly improve caregivers’ hygiene practices and knowledge, often leading to fewer cases of diarrhea. However, the most successful programs were those that used diverse, culturally adapted teaching methods and actively engaged the community. Our findings highlight that while educating caregivers is a vital tool, education alone is not a silver bullet. To achieve lasting reductions in childhood diarrhea, educational programs must be integrated with broader improvements in water and sanitation infrastructure and carefully tailored to the cultural realities of the communities they serve.

## 1. Introduction

Diarrhea is defined as the passage of three or more loose or watery stools per day and can be categorized as acute, persistent, or chronic. Acute diarrhea, which lasts less than two weeks, is often caused by gastrointestinal infections, including viral agents such as rotavirus and norovirus, as well as bacterial and parasitic pathogens [1,2]. It is the third leading cause of death in children under five years of age, particularly in low- and middle-income countries [3]. Globally, approximately 1.7 billion cases of diarrhea occur annually among children, largely due to inadequate sanitation, unsafe water, and poor hygiene practices [2,4]. Preventive strategies include access to safe drinking-water, improved sanitation infrastructure and regular handwashing with soap [5,6]. Moreover, health education programs for parents and caretakers plays a critical role in both prevention and early management of diarrhea episodes. It aims to increase knowledge about hygiene practices, preparation and administration of oral rehydration solutions [5].

However, intervention efficacy varies according to the methodology used and socioeconomic context of the target population [5–7]. A community-based hygiene education study conducted in rural Zaire (now the Democratic Republic of the Congo) showed an 11% reduction in diarrhea incidence [8]. In contrast, another study reported no significant impact on handwashing practices [9]. Given these discrepancies, a comprehensive synthesis of the evidence is needed. This systematic review aims to synthesize evidence from randomized controlled trials evaluating community-based health education interventions for the prevention and management of infectious diarrhea in children under five years of age.

## 2. Methods

### 2.1. Search strategy

This systematic review adhered to the Preferred Reporting Items for Systematic Reviews and Meta-Analyses (PRISMA) [10] framework and followed Cochrane standards for systematic reviews and meta-analyses. A comprehensive search was conducted in MEDLINE/PubMed, EMBASE, Cochrane Library and LILACS for publications up to December 12, 2025, focusing on the Health education in the prevention and management of infectious diarrhea in childhood. Search terms included combinations of “Diarrhea”, “Health Education”, “Child, Preschool” and “Diarrhea/prevention and control”. The full search strategies are presented in S1 Table. In addition to electronic database searches, a manual search of the reference lists of all included studies and relevant reviews was performed to identify additional eligible trials. The study protocol was registered in the International Prospective Register of Systematic Reviews (PROSPERO), bearing the registration number CRD420250643885.

### 2.2. Inclusion and exclusion criteria

Original studies published in any year with full-text availability, regardless of the language of publication, were included. Eligible studies consisted of randomized clinical trials. The population of interest included mothers/caregivers of children under 5 years of age who had access to health education methods (e.g., face-to-face, audiovisual, digital interventions) for the prevention of acute infectious diarrhea. No minimum duration of intervention exposure was predefined, as educational strategies varied substantially across studies. Clinical outcomes of interest included knowledge regarding methods of childhood diarrhea prevention, the number of cases, and adoption of daily hygienic practices following the educational intervention, as well as other outcomes potentially referenced in the articles. Outcome definitions adhered to those provided in the included studies. Cohort studies, case reports, case-control studies, case series, literature reviews, and systematic reviews were excluded. Studies exclusively focused on pharmacological prophylaxis or non-educational preventive interventions were excluded.

### 2.3. Identification and selection of studies

Two authors independently conducted the search, selection, and application of eligibility criteria. The selection process occurred in three stages: (1) removal of duplicates; (2) exclusion of articles that did not meet eligibility criteria based on title and abstract review; (3) and subsequently, full-text review of selected articles with re-application of eligibility criteria to assess their quality and relevance to the proposed objective. Any disagreements between the authors were resolved through discussion and dialogue, with a third author present. The “Rayyan QCRI” tool was used for article selection, and the two researchers were blinded to each other’s decisions throughout the process.

### 2.4. Data extraction

Data extraction was conducted using a predefined data collection form. The following characteristics were collected: study title, reference, country, center, publication year, follow-up period, sample size, and the number of individuals lost to follow-up. Additionally, data on health education methods, topics addressed, implementation and monitoring processes, and participant demographics—including age, participants per group, caregivers’ marital status, education levels, and basic sanitation conditions—were gathered. All outcomes evaluated in the studies were extracted, with an emphasis on health education’s role in preventing and managing childhood infectious diarrhea. Caregiver-reported and other qualitative outcomes were extracted and analyzed according to the definitions, instruments, and criteria adopted in each original study. Two authors independently performed data extraction, and discrepancies were resolved through discussion with a third author.

### 2.5. Risk of bias assessment

The risk of bias assessment included the evaluation of methods for randomization, treatment allocation, blinding, selection and comparability of study groups, and outcome evaluation, conducted at the study level. The following tools were used: the Cochrane Risk of Bias for Randomized Trials (RoB-2) for randomized controlled trials. Judgments were classified as “low risk,” “some concerns,” or “high risk.” These assessments were independently performed by two authors, and discrepancies were resolved through discussion with a third author. No statistical analysis was performed due to the heterogeneity among the included studies.

### 2.6. Data synthesis

Due to heterogeneity in intervention types, follow-up periods, and outcome definitions, meta-analysis and pooled quantitative analyses were not performed. This heterogeneity was particularly related to differences in effect measures and follow-up periods, as well as variability in the content and delivery of educational interventions. Outcomes were reported using different analytical measures, including incidence, prevalence, incidence density, knowledge scores, self-efficacy scales, and behavioral indicators, which limited comparability across studies and precluded meaningful quantitative synthesis. Instead, results were summarized using a narrative synthesis, grouping studies by type of educational intervention and outcome measures.

## 3. Results

### 3.1. Identification and selection of studies

**Fig 1:**
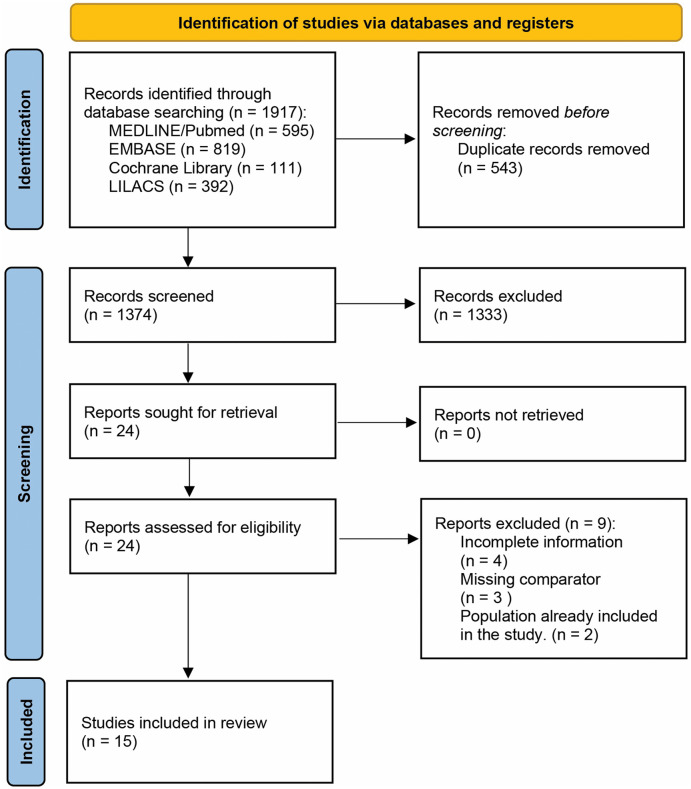
PRISMA [10] flow diagram of study selection.

A total of 1,917 records were initially identified across databases. After removing 543 duplicates, 1,374 studies were screened for title and abstract. 24 studies underwent full-text review, with nine excluded based on predefined eligibility criteria. Ultimately, 15 studies met all inclusion criteria and were included in the qualitative analysis, as illustrated in Fig 1.

### 3.2. Study characteristics

The main characteristics and outcomes of the included studies are summarized in Tables 1 and 2. The final analysis included 15 controlled trials evaluating community-based interventions for the prevention of childhood diarrhea (Table 1). Collectively, these studies enrolled 44,794 caregivers of children between 1985 and 2018. Sample sizes ranged from 51 to 16,301 participants. The studies were geographically diverse, including four conducted in Asia (China, India, Pakistan, Nepal), four in Africa (Gambia, Ghana, Tanzania, Ethiopia), three in Latin America (Brazil, Bolivia, Honduras), and others in South Asia (Bangladesh).

**Table 1:** Baseline characteristics of included studies.

Study	Journal	Period and center	Sample N (%)	Follow-up (months)	Group N (%)	Age (years)
Oles 2024 [11]	BMJ Open	2016 - 2018 Department of Copán in western Honduras	16301	24	Pure Control = 3600 Within-Cluster Control = 7396 Intervention = 5305	Mean 32,9 SD 17.2
Penha 2022 [5]	Maternal and Child Health Journal	2015 Four primary healthcare centers in Brazil	241	2	Control: 60 A: 60 B: 61 AB: 60	NA
Manaseki-Holland 2021 [12]	PLOS Medicine	2015 Central River Region in Gambia	747	32	Control = 377 Intervention = 370	Mean 6-month Control: 27 [22–32] Intervention: 28 [24–32] 32-months Control: 26 [22–30] Intervention: 27 [22–33]
Luo 2019 [13]	Social Science & Medicine	2015 - 2016 Rural China	449	12	Control = 227 Intervention = 222	NA
Ma 2019 [14]	PLOS Medicine	2015 - 2016 Volta Region, Ghana	1956	12	Control = 957 Intervention = 999	Mean Intervention: 23 (SD:16) Control: 22 (SD:15.9)
Guo 2018 [15]	Medicine	2011-2012 Handan, Hebei Province in China	6484	24	Control = 2901 Intervention = 3583	Average: Intervention: 22,46 (SD: 8,5) Control: 22,50 (SD: 8,4)
Hashi 2017 [16]	Preventive Medicine Reports	2015 Jigjiga district, Somali Region, Eastern Ethiopia	1224	6	Control = 596 Intervention = 603	Average: Intervention: 29,7 (SD: NA) Control: 28,5 (SD: NA)
Briceño 2017 [17]	PLOS one	2009 - 2011 Tanzania	5797	24	Handwashing group:1452 Sanitation group: 1433 Combined group: 1431 Control group: 1481	NA
Kapoor 2016 [18]	Indian Journal of Public Health Research & Development	July - November 2014 Chandigarh, India	101	3	Control: 51 (50%) Intervention: 50 (49%)	NA
Lindquist 2014 [19]	The American Journal of Tropical Medicine and Hygiene	May - July, 2010 Cochabamba, Bolivia	1004	3	Control 234 (23%) Intervention A 253 (25%) Intervention B 302 (30%) Intervention A + B 215 (21%)	NA
Nicholson 2014 [20]	Tropical Medicine and International Health	October 2007 - August 2008. Mumbai, India	9675	11	Control 4812 (49%) Intervention 4863 (51%)	NA
Bloch 2013 [21]	Pediatric Emergency Care	April - June, 2010 Academic Pediatric Emergency Department, EUA	104	3	Control 51 (49%) Intervention 53 (51%)	NA
Ansari 2012 [22]	Tropical Journal of Pharmaceutical Research	March 2010 to January 2011, Nepal	632	4	Control 316 (50%) Intervention 316 (50%)	NA
Luby 2004 [23]	JAMA	April 2002 to April 2003 Karachi, Pakistan	4602	12	Control 1483 (32%) Intervention 3119 (68%)	NA
Stanton 1988 [24]	The American Journal of Clinical Nutrition	March - May 1985 Dhaka, Bangladesh	51	12	Control 26 (51%) Intervention 25 (49%)	NA

**Abbreviations**: NA (Not Available); SD (Standard Deviation); RCT (Randomized Controlled Trial); A (Intervention group A); B (Intervention group B); AB (Combined intervention group A + B); Pure Control (Control group in a different cluster); Within-Cluster Control (Control group in the same cluster as intervention); Intervention (Group receiving the evaluated intervention); Control (Group not receiving the intervention).

**Table 2:** Characteristics of the study populations, objectives, and outcomes.

Study	Eligibility Criteria	Objective	Primary Outcomes	Secondary Outcomes
Oles 2024 [11]	Individuals aged 12 years or older, residing in selected villages, who consented to participate and completed the photographic census and baseline survey.	To evaluate the efficacy of a sustained home-based educational intervention to improve practices, knowledge, and attitudes related to maternal and neonatal health.	Diarrhea management, practices related to prenatal care, facility-based births, postnatal care for mothers and newborns, breastfeeding and others.	Knowledge and attitudes related to primary outcomes.
Penha 2022 [5]	Mothers with at least one child under 5 years old, registered at primary healthcare units, and with access to a phone or mobile device.	To evaluate the effects of educational technologies on maternal self-efficacy to prevent childhood diarrhea and reduce its occurrence.	Reduction in the risk of childhood diarrhea. Improvement in maternal self-efficacy scores measured by the EAPDI scale.	NA
Manaseki-Holland 2021 [12]	Children aged 6–24 months and their mothers residing in rural villages. Villages with a population size between 200 and 1,450.	To assess the effects of a community-based intervention promoting safe complementary-food handling practices on reducing infections in children.	Frequency of safe complementary-food handling practices at 6 months.	Reduction in episodes of diarrhea and hospitalizations. Microbiological contamination rates of complementary food and water. Reduction in acute respiratory infections (ARI).
Luo 2019 [13]	Children aged 6–18 months and their primary caregivers residing in rural townships.	Assess the effectiveness of a home-based integrated parenting program on child development and health outcomes.	Reduction in adverse health outcomes (e.g., diarrhea incidence) and improvements in child development (Bayley-III scores).	Parenting practices, dietary changes, caregiver engagement.
Ma 2019 [14]	Households with at least one child under 5 years old in the study area.	Evaluate the impact of CHV home visits on child diarrhea and fever prevalence in Ghana.	14-day diarrhea and fever prevalence among children under 5 years old.	ORS treatment for diarrhea, malaria testing for fever, family planning practices.
Guo 2018 [15]	Children aged 6–40 months, living in selected villages with relatively high HFMD incidence.	To evaluate the effect of hand hygiene education in reducing HFMD incidence.	Incidence of HFMD, improvement in hand-washing habits, reduction in coliform bacteria on hands.	Incidence of respiratory and gastrointestinal symptoms.
Hashi 2017 [16]	Households with at least one child aged 1–59 months, not certified as Model Health Extension Households.	To evaluate the effect of hand washing with soap and WASH educational intervention on the incidence of under-five childhood diarrhea in rural areas.	Longitudinal incidence of diarrhea among children under-five years, measured in episodes per 100 person-weeks.	Bacteriological quality of drinking water at household level (presence of fecal coliforms).
Briceño 2017 [17]	Households with children under 5 years old in the two largest villages in each ward.	Test the interaction between handwashing and sanitation promotion.	Diarrhea prevalence for children under five.	Behavior change including improved latrine construction, levels of open defecation and handwashing with soap.
Kapoor 2016 [18]	Mothers having children <2 years old.	Intervention package for behaviour change practices of mothers to prevent diarrheal episodes in children below 2 years of age.	Improve hand washing practices among mothers.	Behaviour change package on episodes of diarrhea in children.
Lindquist 2014 [19]	Eight peri urban zones southeast economically depressed lacked treated municipal water and sanitation through piped infrastructure.	Evaluate the efficacy of a house-hold level hollow fiber and/or BCC on WASH to reduce the diarrheal disease in children less than 5 years of age.	Diarrheal disease prevalence and stratified diarrhea prevalence.	NA
Nicholson 2014 [20]	Houses with children at least 5 years old in low income urban communities.	Evaluate how a hand washing promotion with provision of free soap, affected illnesses among the children and their families.	Episodes of diarrhea, school absences among target children and episodes of diarrhea among their families.	Episodes of eye infections, vomiting, abscesses or boils, headaches, and earaches.
Bloch 2013 [21]	Caregivers of pediatric patients aged 29 days to 18 years diagnosed with diarrhea.	To determine if adding video discharge instructions affects caregivers’ understanding of their child’s emergency department (ED) visit, plan, and follow-up.	Improve knowledge about the child’s diagnosis, treatment, and follow-up care for diarrhoea.	Caregiver satisfaction with their discharge instructions.
Ansari 2012 [22]	Mothers between the age of 16–40 years, had child/children with diarrhea at the time of enrollment or in the preceding 3–6 months.	Evaluate the effects of educational interventions undertaken at different specified time intervals on mothers’ knowledge, attitude and practice about diarrhea and its management at home.	Comparison of knowledge, attitude and practice between test groups of diarrhoea.	Comparison of knowledge, attitude and practice between test and control during all follow ups.
Luby 2004 [23]	Caregivers had at least 2 children younger than 15 years, at least 1 of whom was younger than 5 years.	Evaluate the effect of promoting household handwashing with soap among children at the highest risk of death from diarrhea.	Incidence density of diarrhea among children, defined as the number of diarrheal episodes per 100 person-weeks of observation.	NA
Stanton 1988 [24]	Caregivers with children aged < 6 years living in 51 slum communities in urban Dhaka.	Evaluate whether an educational intervention was effective in reducing childhood diarrhea.	Rate of diarrhea.	Improving childhood nutritional status.

**Abbreviations**: BCC (behavior change communication); WASH (water, sanitation, and hygiene); NA (not available); HFMD (HAND FOOT AND MOUTH DISEASE).

Follow-up duration exhibited considerable variability, ranging from 2 to 32 months. The majority (n = 9) implemented follow-up periods of 3–12 months, while others maintained 24-months follow-up (Guo et al., 2018 [15]; Oles et al., 2024 [11]), and Manaseki-Holland et al. (2021) [12] extended follow-up to 32 months. Most studies reported caregiver demographics, with ages typically ranging from early adulthood to middle age, although some studies did not specify age ranges (n = 10), and were therefore recorded as “NA” in Table 1.

The majority of studies (n = 11) employed a two-arm design comparing control versus intervention groups, including Luo et al. (2019) [13], Ma et al. (2019) [14], Hashi et al. (2017) [16], and Luby et al. (2004) [23]. Others adopted more complex multi-arm approaches (n = 4), such as Briceño et al. [17], who compared handwashing, sanitation, and combined interventions against a control, while Penha et al. (2022) [5] and Lindquist et al.(2014) [19], evaluated multiple intervention combinations.

The studies included in this review examined population characteristics, intervention objectives, and outcomes related to childhood diarrhea prevention (Table 2). Most interventions targeted caregivers of children under five years of age, with eligibility criteria including child’s age, household location, and caregivers consent to participate.

### 3.3. Interventions and outcomes

The interventions aimed to reduce diarrhea incidence or improve hygiene practices. Oles et al. (2024) [11] and Penha et al. (2022) [5] focused on maternal education, whereas Manaseki-Holland et al. (2021) [12] and Luo et al. (2019) [13] implemented food safety and parenting programs. Several studies, including Ma et al. (2019) [14], Hashi et al. (2017) [16], and Briceño et al. (2017) [17], assessed diarrhea prevalence as the primary outcome, while Guo et al. (2018) [15] and Kapoor et al. (2016) [18] evaluated hygiene-related behaviors.

Secondary outcomes varied across studies, including respiratory infections (Manaseki-Holland et al.,2021) [12], water quality (Hashi et al., 2017) [16], and caregiver knowledge or satisfaction (Ansari et al.,2012 [21]; Bloch et al., 2013 [20]). These caregiver-reported outcomes were based on structured questionnaires or interviews assessing knowledge acquisition, perceived caregiving practices, and satisfaction with the intervention. In contrast, Penha et al. (2022) [5], Lindquist et al. (2014) [19], and Luby et al. (2004) [23] did not report secondary outcomes.

### 3.4. Narrative synthesis of intervention strategies

The educational strategies and corresponding outcomes are synthesized in Table 3. The studies employed diverse health education strategies to prevent and manage childhood diarrhea (Table 3). Delivery methods varied, ranging from traditional face-to-face sessions and printed materials, audiovisual resources, community-based visits, and Behavior Change Communication (BCC) campaigns.

**Table 3:** Characteristics of the health education method used.

Study	Health educational Method	Topics Covered	Implementation	Monitoring	Results
Oles 2024 [12]	Based on Timed and Targeted Counselling with personalized advice using narratives and negotiation with tablets.	Danger signs, and prevention/treatment of diarrhea.	Monthly home visits lasting 1–2 hours by community health worker using audiovisual materials.	Specific questionnaires assessed message retention and ensured consistent delivery of intervention by community health workers.	This study found about 10% increase in the correct identification of appropriate diarrhea treatment methods.
Penha 2022 [5]	Use of an educational video and booklet as pedagogical tools based on Bandura’s self-efficacy theory.	Recognition of diarrhea, personal, environmental and food hygiene, appropriate nutrition, vaccination, actions in case of diarrhea	Interventions conducted at primary healthcare units.	Performed by a trained team using standardized questionnaires and the EAPDI scale at three points.	Diarrhea risk significantly decreased in booklet (A) and booklet and video (AB) groups, with positive associations between maternal self-efficacy and reduced diarrhea.
Manaseki-Holland 2021 [13]	Performing arts and public meetings.	Handwashing before food preparation; Cleaning and drying utensils on clean surfaces; Reheating stored food before feeding. Boiling drinking water for children.	Local health promotion officers, traditional performing artists, and trained community health volunteers.	Home visits with structured checklists conducted by external evaluators.	At 6-month assessment, diarrhea admissions (3% vs. 7%) and incidence (26% vs. 66%) were lower in the intervention group, with sustained reductions at 32-month follow-up.
Luo 2019 [14]	Home-based, biweekly visits by trained community health workers.	Nutrition, hygiene, immunization.	Community health workers trained for 1 week with a structured curriculum and supervised by local officials	App tracking for home visits, unannounced supervisory visits, and caregiver feedback interviews	The intervention reduced diarrhea risk by 8.1 percentage points in the two-week period prior to the survey..
Ma 2019 [15]	Health education through use of visual aids	Prevention of diarrhea, hand hygiene and diarrhea management	CHVs recruited from the local community, trained and tasked with conducting health education and distributing ORS.	Data collectors conducted surveys with caregivers, including questions on health behaviors, CHV activities, and health outcomes.	No significant difference in 14-day diarrhea prevalence was found between intervention and control groups at 6 or 12 months (p = 0.39).
Guo 2018 [11]	Face-to-face training sessions for parents, posters, radio broadcasts, leaflets, village and doctors training parents.	Hand hygiene.	Carried out over two consecutive HFMD epidemic seasons (2011 and 2012) in 64 villages.	Weekly symptom reporting, hand swab collection for coliform bacteria, and knowledge assessment via questionnaires.	The intervention reduced diarrhea incidence from 6.7% to 2.2% and hand contamination from 9.5% to 2.0%, indicating effective impact of hygiene education on diarrheal prevention.
Hashi 2017 [16]	Health education sessions with demonstrations (face-to-face), pamphlets, and use of loudspeakers for key messages.	Hand washing with soap, water storage behavior, latrine availability and use, solid and liquid waste disposal.	12 sessions of health education and demonstrations provided by trained clinical nurse professionals.	Recorded diarrheal episodes, field workers visited households bi-weekly, followed up on water quality, and ensured proper intervention delivery.	The WASH educational intervention reduced diarrhea incidence by 35% among under-five children (IRR = 0.65). Handwashing with soap and hygiene education proved effective in preventing childhood diarrhea in rural Ethiopia.
Briceño 2017 [18]	Marketing interventions and people trained to spread the message of the campaign through face-to-face interactions and help households teaching handwashing.	Handwashing and sanitation.	Trained local FLAs, CLTS facilitators and masons.	At the end of the program.	The interventions improved sanitation and hygiene behaviors, but did not significantly reduce diarrhea prevalence in any group, including the combined intervention.
Kapoor 2016 [20]	Flipbook and pamphlet.	Hygiene & sanitation practices.	Tools and packages were prepared and validated by experts in the field of nursing, community medicine and paediatrics.	Second, third, fifth, eighth and twelfth week after the first visit.	The intervention reduced diarrhea prevalence by more than half, with rates 3.9 times higher in the control group, and significantly improved maternal hygiene and sanitation practices.
Lindquist 2014 [19]	Behavior change-communication.	Family and food hygiene, sanitation, supplementation and water treatments.	Three health technicians, one monitoring and evaluation technician, and one field supervisor received training in adult educational methods, barrier analysis, use of Quality Improvement and Verification Checklists.	Second, third, fifth, eighth and twelfth week after the first visit.	Households using Sawyer PointONE filters had significantly less diarrheal disease compared with the control arm during the intervention period, which was shown by diarrheal prevalence ratios of 0.21 (95% confidence interval [95% CI] = 0.15–0.30) for the filter arm.
Nicholson 2014 [23]	Weekly classrooms and home visits.	Behaviour change aimed to educate, motivate and reward children for HWWS after defecation.	Data collectors were recruited locally and trained on identiﬁcation of illnesses during a 1-day training session led by the study doctor.	Patients were visited twice a week, spaced three or four days apart.	Direct-contact hand washing interventions aimed at younger school-aged children can reduce diarrhea and affect the health of the whole family.
Bloch 2013 [22]	Educational video.	Child’s diagnosis, treatment, and follow-up for diarrhea.	Conducted by medical students, who volunteered as research assistants.	5 question 20 point questionnaire in the ED and, 2–5 days after discharge, another follow-up questionnaire.	Brief video discharge instructions improved caregiver knowledge about diarrhea both in the ED and 2–5 days after discharge compared with written discharge instructions alone.
Ansari 2012 [21]	Educational sessions with text and pictures.	The importance of hygiene, breast feeding, handwashing, use of safe water, sanitation, danger signs of dehydration and correct preparation of ORS.	The interventions were carried out in the form of educational sessions.	Data collection during the second visit (after 1 month) and third visit (after 3 months) by the time of the enrollment.	Intervention was beneficial in improving mothers’ knowledge, attitude and practice regarding diarrhea and its management.
Luby 2004 [17]	Slide shows, videotapes, and pamphlets.	Health problems resulting from contaminated hands and to provide hand washing instructions.	Field workers recruited were extensively trained in interviewing techniques, data recording, approaches to promote handwashing, and measuring and weighing children.	Field workers visited participating households at least weekly for 1 year and asked the mother or other caregiver if the children had diarrhea (3 loose stools within 24 hours) in the preceding week.	Visiting households weekly to provide free soap and encourage hand washing was effective in reducing diarrhea.
Stanton 1988 [24]	Educational messages with posters.	Maternal hand washing before food preparation and sanitation measures.	7 experienced trainers and the 25 volunteer community health workers serving these communities.	Periodic two-week revisits to reinforce the messages.	The intervention reduces rates of childhood diarrhea.

**Abbreviations**: ORS (Oral Rehydration Solution); HWWS (Hand Washing With Soap); CHVs (Community-Based Health Workers); RR (Relative Risk); IRR (Incidence Rate Ratio); AIRR (Adjusted Incidence Rate Ratio); SD (Standard Deviation); CI (Confidence Interval); HFMD (Hand, Foot, and Mouth Disease); BCC (Behavior Change Communication); FLAs (Frontline Activists); CLTS (Community-Led Total Sanitation); ED (Emergency Department); p.p. (Percentage Points).

Three studies implemented home visits as the primary strategy for delivering educational content. Oles et al. (2024) [11], Luo et al. (2019) [13], and Ma et al. (2019) [14] used structured home-based approaches led by trained community health workers or volunteers, supplemented with visual or digital tools. These interventions focused on hygiene practices, diarrhea prevention, breastfeeding, and child nutrition, often incorporating regular monitoring and caregiver feedback mechanisms.

Other studies emphasized group-based educational tools or community engagement strategies. Manaseki-Holland et al. (2021) [12] used performing arts and public meetings, while Penha et al. (2022) [5] applied video and booklet formats based on self-efficacy theory. Hashi et al. (2017) [16] and Guo et al. (2018) [15] conducted face-to-face educational sessions, reinforced by printed materials, community demonstrations, and public broadcasting (e.g., loudspeakers, posters, or radio).

BCC models were central to studies such as Lindquist et al. (2014) [19] and Briceño et al. (2017) [17], which trained local agents to promote hygiene practices and sanitation through structured community interventions. Similarly, Nicholson et al. (2014) [20] applied school- and household-based activities to reinforce child handwashing habits, observing measurable spillover effects on family health.

Bloch et al. (2013) [21] and Ansari et al. (2012) [22] interventions focused specifically on improving caregiver knowledge, using videos, questionnaires, and illustrated sessions to enhance understanding of diagnosis, home care, and signs dehydration signs. Luby et al. (2004) [23] and Stanton et al. (1988) [24] combined education with product distribution, providing printed materials and soap to reinforce hygiene practices.

Overall, 12 studies reported positive outcomes, including reduced diarrhea incidence, improved hygiene behavior, and increased caregiver knowledge. However, a few studies noted that while intermediate behavioral outcomes improved, the interventions alone did not yield significant health impacts (e.g., Briceño et al., 2017 [17]). Monitoring approaches included questionnaires, symptom tracking, unannounced visits, and biological indicators, such as hand swabs or water testing. These outcomes were assessed using caregiver-reported measures related to knowledge, practices, and satisfaction.

### 3.5. Analytical synthesis of the included studies

Demand-side interventions demonstrated robust efficacy in reducing diarrheal disease, with the highest magnitude of effect observed in structural hardware provision. In periurban Bolivia, the installation of hollow fiber water filters was associated with a diarrheal prevalencer ratio of 0.21 (95% CI 0.15 -0.30), representing a 79% reduction in prevalence. Notably, the addition of hygiene BCC did not statistically enhance the filter’s efficacy (PR 0.27, 95% CI 0.22–0.34), while BCC alone produced a non-significant reduction (PR 0.71, 95% CI 0.59–0.86) [17]. Intensive Handwashing with Soap promotion showed high strength of association across urban and rural cohorts. In Karachi, Pakistan, the intervention achieved a 53% lower incidence of diarrhea (95% CI -65% to -41%) [19]. In rural Ethiopia, Hashi et al. reported a longitudinal adjusted incidence rate ratio of 0.65 (95% CI 0.57–0.73), confirming a 35% reduction in diarrhea incidence. Furthermore, a hazard analysis critical control point food safety program in Gambia reduced reported diarrhea with an adjusted relative risk (aRR) of 0.39 (95% CI 0.32 -- 0.48) at 6 months [16]. The benefit of hygiene education extended to viral enteric pathogens. In China, Guo et al. reported that intensive education reduced the incidence of hand, foot, and mouth disease from 4.2% in control groups to 2.1% in intervention groups (x2 = 22.138, p < 0.001). This effect was supported by laboratory findings, with significantly lower coliform contamination detected on hand swabs in the intervention group compared with controls (2.0% vs. 9.45%) [15].

Synthesis of secondary outcomes reveals significant protective effects beyond enteric pathologies. Handwashing promotion in Mumbai, India, was associated witha 15% reduction in episodes of acute respiratory infection (ARI) episodes (95% CI -30% to -8%) and a 46% reduction in eye infections (95% CI -58% to -31%) among 5-year-olds children [20]. Similarly, the Gambia trial observed an aRR for ARI of 0.67 (95% CI 0.53 - 0.86). These data suggest that the mechanism of action for hand hygiene extends beyond the fecal-oral route to respiratory and ocular pathogen transfer pathways [12].

Integrated programs demonstrated the ability to alter cognitive trajectories without necessarily impacting distal physical growth outcomes. In rural China, an integrated home-visitation program improved cognitive development by 0.24 standard deviations (SD) (95% CI 0.04–0.44 SD, p = 0.01). However, no significant effects were found for motor or social-emotional development [13]. A critical finding across the literature is that reductions in morbidity do not necessarily translate into measurable gains in nutritional status. For example, in Bangladesh an intervention achieved a 22% protective efficacy against diarrhea (incidence density ratio of 0.78, 95% CI 0.74–0.83). Despite this reduction, children in both intervention and control groups exhibited identical patterns of weight gain and remained at 76% of the NCHS standard for weight-for-age. This underscore the complexity of growth outcomes and suggests that factors such as food security, dietary quality and metabolic stressors may limit the impact of hygiene-only interventions on nutritional recovery [24].

Educational technologies reliably improved knowledge scores and maternal self-efficacy (MSE). In the United States, Bloch et al. demonstrated that a 3-minute video intervention significantly outperformed written instructions, with higher knowledge scores both immediately (12.2 vs. 8.9) and at follow-up (11.1 vs. 7.8) [21]. In Brazil, the use of combined video and booklets significantly improved MSE scores, with the risk of childhood diarrhea in the combined group (AB) plummeting from 8.5 to 1.1 over the follow-up period [5]. In Honduras, counseling led to significant shifts in primary care practices. Targeted parents were 16.4% more likely to seek professional newborn checks within 3 days (95% CI 3.1%–29.8%, p = 0.016) and 19.6% more likely to avoid traditional umbilical cord wrapping (95% CI 4.2%–35.1%, p = 0.013) [11].

The strength of the reported associations is moderated by several identified confounders that were not fully controlled in the primary studies. In Ghana, Ma et al. (2019) noted a district-wide cholera outbreak triggered supply-side interventions (e.g., water tablets) that caused diarrhea prevalence in the control group to drop from 20.1% to 7.0%, potentially masking the true effect of volunteer visits (overall RR 0.73, 95% CI 0.37–1.45). Subgroup analysis in the Ghana trial identified that health benefits were only statistically significant when implementation met specific fidelity thresholds: ≥ 70% community coverage and ≥30 minutes per visit (Diarrhea RR 0.23, p = 0.003) [14]. In Bolivia, the extreme baseline contamination of tanker truck water (71.8% of households) acted as a structural confounder that rendered behavioral education ineffective without the physical barrier of a filter. In the end, most studies relied on maternal 7-day or 14-day recall, and several lacked blinding between the delivery and data collection teams, introducing potential social desirability bias [19].

## 4. Discussion

This systematic review highlights that community-based educational interventions targeting caregivers of children under five years old are associated with effectiveness in preventing childhood diarrhea, though the overall impact was modest. There was a methodological diversity, both in educational strategies and geographic contexts, across the 15 studies, illustrating the remarkable adaptability of these interventions to various socioeconomic and cultural settings. This heterogeneity limited outcome comparability and generalizability.

Some intervention characteristics were associated with more favorable outcomes. Periodicity and reinforcement strategies, for example, appear critical for sustaining behavioral change. In the study by Manaseki-Holland et al. (2021), the intervention included multiple campaign visits over 25 days, a follow-up reinforcement visit after five months, and informal involvement of community volunteers. This structure led to a 60% reduction in self-reported diarrhea cases at six months. However, these findings should be interpreted in light of the study design and reliance on self-reported outcomes, and although the effects weakened over time, improvements were still partially sustained at 32 months—even without programmatic support [12]. Another important factor is the diversity of educational strategies used. Multicomponent approaches—especially those combining structured home visits, audiovisual materials, and BCC campaigns—were consistently associated with reduced diarrhea incidence and improved hygiene practices. These findings suggest that integrated and culturally appropriate interventions, when delivered with sufficient intensity and continuity, may lead to more sustainable outcomes in child health.

These findings align with a robust and expanding body of evidence reporting the efficacy of community-based health education initiatives, particularly those employing participatory approaches and culturally adapted methods. The reviewed studies demonstrate a consistent pattern of positive outcomes, including improved hygiene practices, enhanced caregiver knowledge, and in some cases, reduced diarrhea incidence. While effect size varied across interventions, the results align with previous evidence supporting participatory and culturally sensitive health education approaches [4, 25].

Furthermore, the insights gained echo the findings of Bhutta et al. (2013), whose work on community engagement in pneumonia management—although focused on a different clinical condition—demonstrates the transferable value of adaptable, culturally embedded interventions in improving child health outcomes [26]. This parallel supports the potential relevance of community-based approaches across different child health domains, particularly when local realities are considered in program design.

However, an important consideration in the interpretation of these findings is the extent to which interventions are culturally adapted to the local context. Several studies included in this review—such as those by Penha et al.(2022), Luo et al.(2019), Ma et al.(2019), Kapoor et al.(2016), Bloch et al.(2013), Ansari et al.(2012), Luby et al.(2004), and Stanton et al.(1988)—do not explicitly report whether or how cultural norms, local knowledge, or traditional practices were incorporated into the design or implementation of their educational strategies [5,13,14,23,18,22,21,24]. In these cases, interventions appear to have followed more standardized, externally designed formats, with limited reference to participatory or context-specific adaptations. This may represent a limitation in terms of community engagement, local relevance, and long-term sustainability. Community-based knowledge, including traditional approaches to diarrhea management and prevention, represents a potentially valuable asset that is seldom integrated into program design. Greater emphasis on cultural contextualization and local participation could enhance the relevance, uptake, and overall effectiveness of future educational interventions.

Crucially, interventions characterized by direct engagement—such as structured home visits, the use of context-specific visual communication tools, and dialogical educational sessions—demonstrated superior effectiveness compared to more passive or didactic strategies. Programs such as those implemented by Oles et al. (2024), Luo et al. (2019), and Ma et al. (2019) [11,13,14]. illustrate the transformative potential of pedagogical approaches that are not only linguistically accessible but also culturally resonant and behaviorally relevant. In parallel, strategies that involved the training and mobilization of community health workers—exemplified by the interventions of Lindquist et al. (2014) and Briceño et al. (2017) —were instrumental in fostering long-term behavior change and reinforcing trust in local health systems [17,19].

Moreover, this review is the detailed synthesis of educational methods employed across studies. Interventions ranged from traditional in-person sessions, illustrated booklets, and radio broadcasts to modern digital and video-based formats. Structured home visits by trained community health workers emerged as particularly effective, enabling tailored advice, trust-building, and behavior reinforcement (Luo et al., 2017; Oles et al., 2010) [10,11]. Group education using theater, public meetings, or community games, as observed in studies like Manaseki-Holland et al. (2021), fostered peer support and community engagement. This diversity in delivery modes highlights that educational interventions are not one-size-fits-all but benefit from contextual adaptation and multimodal strategies (Tomlinson et al., 2013; George et al., 2019) [12,25,27].

To our knowledge, this is the first systematic review to focus specifically on community-based educational interventions aimed at preventing childhood diarrhea through caregiver engagement—addressing a critical and previously underexplored gap in the literature. While earlier reviews have assessed broader water, sanitation, and hygiene (WASH) strategies (Ejemot-Nwadiaro et al., 2015; Freeman et al., 2014), none have centered exclusively on educational components directed at caregivers, who have an important influence on the early recognition, prevention, and management of diarrheal disease in children under five [28,29].

### 4.1. Biases and limitations

**Fig 2:**
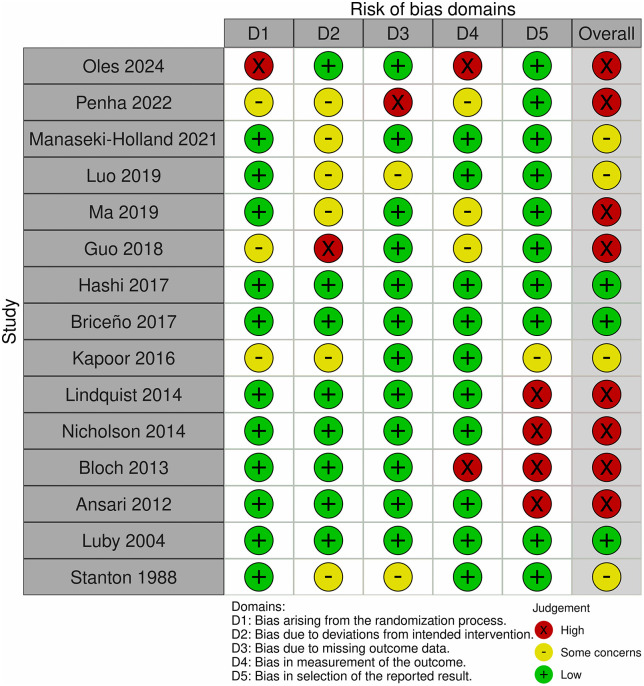
Risk of Bias Graph for the Different Clinical Trials Included in the Systematic Review.

The risk of bias analysis indicated that some studies had a high risk in domains such as inadequate randomization, lack of blinding, and substantial loss to follow-up (Fig 2). Studies that failed to clearly describe randomization methods or to implement strategies to minimize performance and detection bias tend to yield less reliable results, compromising internal validity and the strength of the synthesized evidence. Oles et al. (2024) and Penha et al. (2022) were particularly notable for high risk related to randomization and deviations from intended interventions, including the use of self-reported data and potential lack of blinding [5,11]. Penha et al. (2022) also showed high risk due to missing outcome data, with follow-up losses that may have influenced the results [5]. Similarly, Lindquist (2014) and Nicholson (2014) exhibited high risk in outcome measurement due to reliance on caregiver-reported morbidity without clinical verification, increasing the possibility of detection bias [19,20].

Other studies, such as Luo et al. (2019), Ma et al. (2019), and Manaseki-Holland et al. (2021), were classified as having “some concerns,” especially regarding allocation concealment and outcome measurement [12–14]. Conversely, the direction and strength of the observed associations were more consistent when supported by studies classified as having Low Risk of Bias. For example, Luby (2004) and Hashi (2017) provided higher-certainty evidence that intensive handwashing promotion and consistent WASH messaging were associated with reductions in diarrhea incidence ranging from 35% to 53% in high-risk environments. Studies categorized as having “some concerns,” such as Manaseki-Holland (2021) and Luo (2019), also demonstrated favorable short-term reductions—such as the 60% decrease in self-reported diarrhea observed in The Gambia following multiple reinforcement visits—although these findings require cautious interpretation due to methodological constraints.

Conversely, findings from studies with High Risk of Bias should be interpreted with greater caution, particularly when based on self-reported outcomes or in the presence of incomplete follow-up data, as these factors may overestimate intervention effects. These findings highlight the need for caution when interpreting the effectiveness of the interventions.

Despite consistent behavioral improvements, several interventions did not translate into clear clinical benefits, suggesting that structural determinants, such as inadequate sanitation and limited access to clean water, may attenuate the isolated effect of educational strategies (Freeman et al., 2014; Luby et al., 2006) [23,29]. This contextual dependency represents an important limitation when interpreting effectiveness estimates across heterogeneous settings. Environmental and district-level confounders also influenced observed impacts. In Ma (2019), for instance, a district-wide cholera outbreak triggered concurrent supply-side interventions in the control group, potentially masking the true effect of the educational strategy. In addition, incomplete reporting of participant characteristics, particularly caregiver age, in several included studies restricted the assessment of population comparability and may have contributed to observed clinical heterogeneity. Together, these factors limit causal inference and underscore the need for cautious interpretation of pooled qualitative findings.

## 5. Conclusion

This systematic review concludes that community-based educational interventions targeting caregivers of children under five years of age seems to be associated with improvements in hygiene-related behaviors, caregiver knowledge, and, in some settings, reductions in childhood diarrhea incidence. However, the strength and consistency of these effects varied across studies and were influenced by methodological quality, intervention intensity, and contextual factors.

Such recommendations can, in turn, inform the refinement and optimization of educational tools and strategies aimed at improving hygiene practices and preventing diarrhea in children. To maximize their public health impact, these tools must be integrated into broader health systems and WASH (Water, Sanitation, and Hygiene) initiatives, while being continuously adapted to local realities.

From a public health perspective, these findings reinforce the role of caregiver-focused educational strategies as complementary tools within broader child health and WASH policies. By improving caregiver awareness, care-seeking behaviors, and adherence to recommended hygiene practices, such interventions may contribute to reducing preventable morbidity in vulnerable populations. However, their impact depends on integration with health system capacity, availability of essential supplies, and long-term monitoring to ensure sustainability and safety.

Importantly, the risk-of-bias assessment indicates that although caregiver education represents a relevant and potentially effective strategy, the overall certainty of the current evidence remains uneven. Variability in study design, outcome measurement, and reporting quality limits causal inference and generalizability. Furthermore, a formal assessment of the overall certainty of the evidence using the GRADE approach was not carried out, and this represents an important limitation of the review, as it impacts the strength of recommendations that can be drawn from the conclusions. Therefore, future research should prioritize well-designed randomized controlled trials with standardized protocols, objective compliance metrics, and longer follow-up periods to mitigate recall and performance biases and to strengthen the robustness of the evidence base.

Ultimately, the effectiveness and sustainability of these interventions depend on their ability to incorporate socio-cultural dimensions and to be embedded within adequately resourced health and sanitation systems. Bridging the gap between evidence-based practices and culturally rooted caregiving habits is crucial for fostering mutual learning and co-creating health solutions that are both impactful and locally meaningful.

## Supporting information

S1 Table Detailed and reproducible search strategies used for each database. Comprehensive search strategies for MEDLINE (PubMed), Cochrane Library, EMBASE, and LILACS, including controlled vocabulary terms (MeSH/Emtree) and free-text terms, with Boolean operators and filters applied.
(DOCX)

S1 PRISMA Checklist PRISMA 2020 Checklist. Completed PRISMA 2020 checklist indicating the location of each reporting item within the manuscript.
(DOCX)
