# Supplementary material for: Health education in the prevention and management of infectious diarrhea in childhood: A systematic review of randomized controlled trials
Source: PLoS Negl Trop Dis. 2026 Jul 23;20(7):e0014442. doi: 10.1371/journal.pntd.0014442 (PMC13423171; doi:10.1371/journal.pntd.0014442)
Supplement: S1 Table — Comprehensive search strategies for MEDLINE (PubMed), Cochrane Library, EMBASE, and LILACS, including controlled vocabulary terms (MeSH/Emtree) and free-text terms, with Boolean operators and filters applied. (DOCX) [file pntd.0014442.s001.docx]

***S1 Table . Detailed and reproducible search strategy used for each database***

| ***Database*** | ***Search strategy*** |
| --- | --- |
| ***PubMed (MEDLINE)*** | *("Diarrhea/prevention and control"[Mesh] OR "Diarrhea/therapy"[Mesh] OR diarrhea[tiab]) AND ("Health Education"[Mesh] OR "health education"[tiab] OR "health promotion"[tiab] OR "community education"[tiab]) AND ("Child, Preschool"[Mesh] OR "Infant"[Mesh] OR "children under 5 years"[tiab] OR "under five"[tiab])* |
| ***Cochrane Library*** | *("Diarrhea/prevention and control" OR "Diarrhea/therapy" OR diarrhea) AND ("Health Education" OR "health education" OR "health promotion" OR "community education") AND ("Child, Preschool" OR "Infant" OR "children under 5 years" OR "under five")* |
| ***Embase*** | *('diarrhea'/exp OR 'diarrhea prevention'/exp OR 'diarrhea treatment'/exp OR diarrhea:ti,ab) AND ('health education'/exp OR 'health promotion'/exp OR 'community education':ti,ab OR 'health education':ti,ab) AND ('preschool child'/exp OR 'infant'/exp OR 'children under 5 years':ti,ab OR 'under five':ti,ab)* |
| ***LILACS*** | *(diarreia OR diarréia OR "Diarréia/prevenção & controle" OR "Diarréia/terapia") AND ("Educação em Saúde" OR "Promoção da Saúde" OR "Educação Comunitária" OR educação OR "health education" OR "health promotion" OR "community education") AND ("Criança Pré-Escolar" OR "Lactente" OR "Crianças menores de 5 anos" OR "menores de cinco anos" OR "child, preschool" OR "infant" OR "children under 5 years" OR "under five")* |

*In addition to electronic database searches, manual searches of reference lists of all included studies and relevant reviews were performed to identify additional eligible trials. All records identified through manual searching were screened using the same predefined eligibility criteria applied to those retrieved from electronic databases.*
